# The case for genetic monitoring of mice and rats used in biomedical research

**DOI:** 10.1007/s00335-012-9444-9

**Published:** 2013-01-12

**Authors:** James R. Fahey, Hideki Katoh, Rachel Malcolm, Ana V. Perez

**Affiliations:** 1 Laboratory Animal Health Services Department, The Jackson Laboratory, 600 Main Street, Bar Harbor, ME 04609 USA; 2 Hamamatsu University School of Medicine, Hamamatsu, Shizuoka Prefecture 431-3125 Japan; 3 Genetic Sciences and Compliance, Taconic Farms Inc., One Hudson City Centre, Hudson, NY 12534 USA

## Abstract

Currently, there is the potential to generate over 200,000 mutant mouse strains between existing mouse strains (over 24,000) and genetically modified mouse embryonic stem cells (over 209,000) that have been entered into the International Mouse Strain Resource Center (IMSR) from laboratories and repositories all over the world. The number of rat strains is also increasing exponentially. These mouse and rat mutants are a tremendous genetic resource; however, the awareness of their genetic integrity such as genetic background and genotyping of these models is not always carefully monitored. In this review, we make a case for the International Council for Laboratory Animal Science (ICLAS), which is interested in promoting and helping academic institutions develop a genetic monitoring program to bring a level of genetic quality assurance into the scientific interchange and use of mouse and rat genetically mutant models.

## Background

The explosive growth of biomedical research over the past several decades has provided immense insight into the role of genes in human health and disease (Peters et al. 2007; Svenson et al. 2007). One consequence of this spectacular advancement in biology has been a concomitant expansion in the number, complexity (Steuber-Buchberger et al. 2008; Yu and McMahon 2006), and diversity of genetically defined animal models developed as research tools with which to study genes and their function. During the last 25+ years, improvements in technologies that allow genetic manipulation of the mouse genome, such as mouse oocyte pronuclear injections (Gordon and Ruddle 1983), targeted mutagenesis (Doetschman et al. 1988; Pevny et al. 1991), and ENU mutagenesis (Sabrautzki et al. 2012), have allowed the production of thousands of genetically modified mouse strains. Development of targeted rat mutant stocks using similar techniques, including ENU mutagenesis (Kuramoto et al. 2011; Mashimo et al. 2008; van Boxtel et al. 2010), has provided scientists with options for development of new rat strains. Nonetheless, large-scale transgenic rat strain development has lagged behind that of mice primarily because until very recently (Li et al. 2008, 2009; Liao et al. 2009), a lack of induced pluripotent stem cells or embryonic stem (ES) cells required for targeted mutagenesis. However, these recently introduced stem cells and germline cell lines, in conjunction with the introduction of zinc finger nucleases and TALE nucleases, have enabled the successful development of new rat models and genome editing of rats (Cui et al. 2011; Geurts et al. 2009; Geurts et al. 2009; Tong et al. 2012; van Boxtel and Cuppen 2010). It is important to point out that these genomic manipulation technologies, by virtue of their adaptability, will allow for rapid development of new mouse and rat models, including the sequential modification of existing strains. These technologies will enable a considerable increase in the numbers of genetically modified animals.

Genetically defined mouse and rat models, including inbred, recombinant inbred, consomic, congenic, outbred, and genetically modified strains, have now become readily available to research institutions through the world [see The International Mouse Strain Resource (IMSR), http://www.findmice.org/index.jsp]. This trend is likely to continue as additional genetically defined rodent models are developed to answer questions about specific gene functions and gene–gene interactions [see, e.g., http://grants.nih.gov/grants/oer.htm, PA – 07-336, Development of Animal Models and Related Biological Materials for Research (R21)]. In addition, several funded projects have generated thousands of mutant mice, including the NIH Knockout Mouse project (KOMP, http://www.genome.gov/15014549) and the more ambitious mouse phenome project, which includes the goal of establishing baseline phenotypic data for the most common inbred mouse strains (http://www.ncbi.nlm.nih.gov/pubmed/15619963). To be sure, this effort is based on the genetic integrity of the mice that are being characterized. Therefore, to be able to compare and interpret experimental information, it becomes critical to have genetically well-characterized mouse and rat mutants. This trend of increasing the availability of genetically defined rodent strains, however, also begs the question of whether the dramatic increase in rodent models has been accompanied by a simultaneous increase in effort to monitor the genetic quality of these animals. In other words, do scientists, veterinarians, animal care personnel, and animal facility managers know that the specific strains of research animals they develop, use, care for, and house are genetically sound and truly representative of their assumed genotype or genetic background? With greater numbers of research mice and rats of profound genetic diversity being utilized and an increase in the exchange of genetically modified rodent models between scientists and animal facilities, what are the chances that a genetic contamination is present in these animals from an error in genetic modification, or a breeding error? What are the chances that genetic drift, an accumulation of mutations over time, has occurred even though the research animals look no different from their progenitors?

Another important consideration that needs to be addressed when designing an experiment is the choice of the genetic background in which the mutation will be maintained. Phenotype variation due to genetic background is well documented (Threadgill et al. 1995; Sibila and Wagner 1995). It is critical to maintain the genetic background; this refers not only to different inbred genetic backgrounds but also to whether a mixed or outbred background is needed. Some mutations are able to survive only in mixed or outbred backgrounds. The critical issue when outbred or mixed stocks of mice and rats are used versus inbred strains lies in the different ways outbred or mixed stock needs to be maintained versus an inbred strain. The methods for breeding inbred and outbred stocks and strains are completely different. Maintaining heterozygosity is critical for breeding outbred mice and rats, whereas homogeneity of the genetic background is the principal emphasis in breeding inbred strains of mice and rats. Therefore, genetic monitoring of outbreds is quite different than genetic monitoring of inbreds.

The International Council for Laboratory Animal Science (ICLAS) is interested in helping research institutions answer the question of genetic identity of their animals. For many years the ICLAS has sponsored programs dedicated to the promotion of high-quality animals for use in research. In particular, the ICLAS has focused on the ethical treatment of animals, animal health, and genetic quality (Hedrich 1990; Nomura et al. 1984). In this regard, the ICLAS recently developed a program called the “ICLAS Network for Promotion of Animal Quality in Research” (or ICLAS Animal Quality Network), whose specific aim calls for the development of a genetic monitoring program on an international scale. The focus of this genetic monitoring program is to assure the reproducibility of experimental results by using genetically defined animals, which, like all defined reagents used in an experiment, can directly impact the results. The negative impact of using poorly defined animals is very real and can be manifested as research data rendered meaningless or unrepeatable due to findings with genetically contaminated animals. There is also the loss of scientific reputation and the waste of research dollars that result from discarded inaccurate data, and there is often the need to repeat studies with genetically defined animals. The ICLAS scientists and member institutions recognize the profound importance of genetically defined rodents as models of scientific research and the value of these models as analogs of human biological and genetic processes. This document reviews the importance of genetic monitoring programs for rodent-based research and provides a strategy for how to begin genetic monitoring via the ICLAS Animal Quality Network.

## How do you establish and maintain rodent breeding colonies of high genetic quality?

Several substantive reviews have been written that discuss the basic requirements for breeding programs designed to provide high-quality inbred strains of mice and rats for research (Fox et al. 2007; Hedrich 1981; Katoh 2006; Poiley 1960). In consideration of this topic, it is also important to recognize the use of outbred stocks (Chia et al. 2005; Kagiyama 1999). It is worth reiterating some of the important points described in these reviews that pertain to traditional strategies for maintaining genetically defined inbred or outbred (Nomura 1999) stocks of mouse and rat colonies and testing their genetic background. Foremost among these critical points is that animal husbandry practices, including well-established institutional guidelines for colony management, are the primary means used to establish and maintain inbred and outbred stocks of mice and rats. They are also the principal means by which breeding errors that lead to genetic contamination are prevented. Thus, initial training and periodic training updates of animal husbandry personnel provide the foundation for the maintenance of genetically defined animals. Components of training programs vary by institution but must minimally include information on husbandry and breeding methods, strain nomenclature, strain characteristics (e.g., behavior, coat color, fecundity), and, perhaps most crucially, careful record-keeping methods for breeding colonies. Well-trained animal care personnel, by virtue of their familiarity with rodent strain characteristics, are often quick to recognize anomalies in mouse and rat strains resulting from genetic contamination, phenotypically apparent mutations, or other perturbations, including infectious diseases. It is for this reason that commercial rodent breeders are particularly reliant on the observant eyes of experienced animal husbandry personnel to bring attention to changes in the characteristics of inbred mouse and rat strains. It is also why commercial rodent breeders invest substantial time and money in the training of their animal care personnel. Further, it is essential for the maintenance of rodent stocks or strains that efforts be undertaken to minimize substrain divergence and genetic drift in inbred or outbred colonies, that is, that undetected spontaneous mutations become fixed in an inbred colonies over time, or that in outbred colonies random genetic drift potentially fixes unwanted homozygous alleles. To preserve the genetic stability of these colonies, it is recommended that either new breeding stock be obtained periodically from a commercial supplier, or the number of generations produced by the breeding stock be minimized prior to regularly scheduled refreshment of the stock with cryopreserved embryos or gametes. This routine maintenance of the colonies will assure that these valuable resources change as little as possible over time, thereby assuring that the biological research performed with these rodent models is as accurate and reproducible as possible (Taft et al. 2006).

## How do you confirm the identity of inbred rats and mice?

Even with the best trained animal care personnel and a genetic stability program, it is still appropriate and necessary to complement this surveillance with laboratory methodologies that objectively identify genetic homogeneity within established inbred strains of mice and rats. The current paradigm for genetic monitoring of inbred strains of mice and rats at commercial rodent breeders and many academic institutions is based on various molecular methods of DNA sequence identification for confirming the genetic identity of the animals in question (Basta et al. 2004; Bryda and Riley 2008; Petkov et al. 2003, 2004). Nevertheless, this is a relatively recent development and was preceded by the use of biochemical, immunological, phenotypic, and morphometric strain characterizations, all of which are still valid methods for confirming the genetic identity of inbred rodent strains. The most important criterion in the development of a reliable genetic monitoring program is formally establishing the genetic characteristic(s) to be monitored. In other words, what genetic attribute or set of attributes can be measured with confidence via cost effective methods to provide a measure of the homogeneity of the genetic background of animals being monitored? Further, it is essential that the monitoring process be simple to understand and implement. There are precedents for making this decision based on published methods of genetic monitoring. For example, Hedrich (1981) suggested a combination of testing methods such as biochemical, immunological, and morphological for standardized monitoring of breeding colonies of mice, with the extent of testing based on the hierarchical breeding level of the animals. In this scheme, foundation stocks of mice would be tested for a greater range of mono- and polygenetic markers than would pedigree expansion stocks. Also, it is at the early stages of this hierarchy that the most harm and downstream expense would be incurred should there be an error that, say, leads to genetic contamination. This stands to reason because all breeding downstream would be affected by genetic changes (mutations, contamination) in the foundation stocks as opposed to the pedigree expansion stocks.

It should be noted that Hedrich’s suggested methods of genetic monitoring are comprehensive and robust but were made prior to the availability of large-scale molecular testing for genetic identification. More recently, Katoh (2006) described an approach to genetic monitoring that incorporates sets of markers into levels or profiles selected for use based on the purpose of the monitoring. For example, the Genetic Profile encompasses 19 individual biochemical and immunological markers for certification of the genetic quality of an inbred mouse strain. This test profile will detect gross genetic contamination but cannot identify genetic drift. Two additional profiles, the Monitoring Profile and the Critical Subset Profile, utilize sets of limited markers for confirmation of mouse strain genotypes and for verification of the genotype of inbred strains within a particular room or facility. This latter profile is used, for example, to confirm genotypes among several albino mouse strains housed within the same area since the profile will easily uncover mistakes in breeding. Katoh (2006) also describes the development of a genetic monitoring kit that supplies the basic tools necessary to get a genetic monitoring program underway, but which can also be used to educate interested personnel on how to identify genetic markers in mice and rats. This kit is based on phenotypic and immunological strain identification and does not include molecular identification methods.

The aforementioned molecular methods of genetic identity monitoring, e.g., simple sequence length polymorphism (SSLP) or microsatellite genotyping (Basta et al. 2004) and single nucleotide polymorphism (SNP) genotyping (Petkov et al. 2003), offer several advantages over traditional biochemical, immunological, and phenotypic methods of genetic identification. First, both methods can be performed with DNA from tissue samples from a tail biopsy so that euthanasia of the subject mouse or rat is not required. Second, both methods are PCR-based, making them easy to acquire and master by any laboratory performing routine PCR work. Third, both methods are sensitive, reproducible, and can be scaled up for testing large numbers of samples without excessive costs. SNPs have additional advantages, however, when compared to SSLP markers for genetic identity testing. SNPs are a more abundant genetic variation marker than SSLP markers and thus offer a more robust means of differentiating mouse strains (Petkov et al. 2004). Furthermore, SNP testing has been shown to be a faster, more efficient, and less expensive genotyping method compared to SSLP testing (Peters et al. 2007). In addition, SSLPs have a higher rate of mutation than SNPs (Dallas 1992; Kruglyak et al. 1998). Irrespective of which molecular method, SNP or SSLP testing, is chosen for genetic identification of mice or rats, both of these modern DNA-based technologies are an excellent way of establishing a genetic monitoring program that will provide reliable results.

## How do you confirm the identity of outbred rats and mice?

Unlike inbreds, outbred mice and rats have heterozygous genomes. This gives them some of the phenotypic characteristics that differentiate outbred stocks from inbred strains, i.e., fecundity and larger litter sizes, making them easier to breed. At the same time, this genetic heterozygosity makes outbred stocks prone to random genetic drift, primarily as a result of genetic bottlenecks when a small number of animals are used either while breeding or during derivations. Outbreds are used for different purposes than inbreds and the conservation of genetic variability is important. Therefore, outbred stocks are bred in such a way as to maintain the maximum genetic variability of the stock (Poiley 1960). Commercial breeders usually maintain very large colonies that assure a large number of breeders. The challenge becomes how to establish an effective genetic monitoring program for such a diverse population and for identification of the important genetic factors that one should monitor in outbred colonies. Nomura (1999) expressed the need for establishing global rat outbred genetic standards; these standards have been accepted by most commercial vendors. In addition to an appropriate breeding program that assures genetic variability, this program should be able to maintain the genetic profile of the stock by testing a set of genetic polymorphisms. The same considerations outlined above for inbred strains regarding the methodology used for testing are applicable for outbred strains. The only difference is that for outbreds a genetic profile of heterozygous markers needs to be established for each particular stock and this genetic profile needs to be monitored throughout the existence of the colony.

## What can institutions that are not commercial or central rodent distribution centers do to train personnel and implement a genetic monitoring program?

The ICLAS Animal Quality Network program is currently developing the means by which any institution that uses mice and rats as research models can develop the in-house capability to perform genetic quality monitoring of their rodent colonies. However, ICLAS also recognizes that there is a spectrum of capabilities among different research institutions with regard to their capacity to establish, maintain, and monitor colonies of genetically defined animals. Therefore, training and education are of paramount importance in this effort. For this reason, the Animal Quality Network program will promote training and education, first to cover the essentials of good colony management, including basic genetics, understanding of rat and mouse strain characteristics, and breeding propensities, and familiarity with methods of cryopreservation and record keeping. Mastering these core fundamentals is a prerequisite to the introduction of a formal genetic monitoring program, for which ICLAS will also promote training and education curricula that teach the methodologies available for genetic monitoring of rodents. Finally, the Animal Quality Network program will establish a self-assessment genetic monitoring program for interested member institutions. The concept of this program will be modeled on the current ICLAS Performance Evaluation Program (PEP) that provides participating laboratories with a mechanism to self-assess their health monitoring and accuracy of testing for rodent pathogens. Similarly, the self assessment genetic monitoring program will enable institutions to determine whether their testing methods accurately identify the genetic background of the rodent strains being tested. The program will start with trials involving those research facilities currently performing rodent genetic monitoring and will be opened later to other institutions as they become confident in their capabilities. ICLAS will periodically publicize the means to access the Animal Quality Network program for genetic monitoring through this journal and other laboratory animal science journals, seminars, and special events as the program becomes firmly established.

## Conclusions

Availability of a variety of molecular biology technologies gives relatively easy access to different types of DNA testing that can be used in the establishment of a genetic monitoring program “in house.” A genetic monitoring program is necessary to safeguard the genetic quality of the mice/rats that are housed in a barrier. This genetic monitoring program is dependent on the type of mouse or rat model being bred and the genetic composition of other mouse/rat models in the particular barrier. Therefore, a genetic monitoring program needs to be inclusive of the genetic risks that other strains or models that are cohoused pose to the particular barrier. In order to achieve this, ICLAS, through its Animal Quality Network Program, is interested in (1) promoting training and education to increase awareness of the importance of genetic quality and genetic monitoring, (2) providing advice and guidance on genetic quality testing, and (3) establishing a self-assessment genetic monitoring program for interested institutions.
